# Serum Acrolein and Peripheral Arterial Stiffness in Non-Dialysis Chronic Kidney Disease: A Cross-Sectional Study

**DOI:** 10.3390/toxins18060246

**Published:** 2026-05-29

**Authors:** Ho-Hsiang Chang, Yu-Li Lin, Chi-Chong Tang, Chiu-Huang Kuo, Chih-Hsien Wang, Bang-Gee Hsu

**Affiliations:** 1 Division of Nephrology, Hualien Tzu Chi Hospital, Buddhist Tzu Chi Medical Foundation, Hualien 97004, Taiwan; 2 School of Medicine, Tzu Chi University, Hualien 97004, Taiwan; 3 Institute of Medical Sciences, Tzu Chi University, Hualien 97004, Taiwan; 4 School of Post-Baccalaureate Chinese Medicine, Tzu Chi University, Hualien 97004, Taiwan

**Keywords:** acrolein, cardio-ankle vascular index, peripheral arterial stiffness, chronic kidney disease, urine protein-to-creatinine ratio

## Abstract

Background: Arterial stiffness is a major predictor of cardiovascular-related mortality in chronic kidney disease (CKD). While acrolein—an endogenous reactive aldehyde that accumulates with declining renal function—may be linked to vascular injury, its association with high peripheral arterial stiffness (HPAS) remains unclear. Methods: This cross-sectional study used the cardio-ankle vascular index (CAVI) to examine the association between serum acrolein levels and HPAS (CAVI ≥ 9.0) in 204 adults with non-dialysis CKD stages 3–5. Results: HPAS was identified in 90 patients (44.1%) and was associated with a higher prevalence of diabetes (*p* = 0.047), older age (*p* = 0.023), higher spot urine protein–creatinine ratio (UPCR, *p* = 0.046), higher serum fasting glucose (*p* = 0.041), higher interleukin-6 (*p* = 0.025), and higher acrolein (*p* = 0.008). In multivariable logistic regression analysis, serum acrolein (odds ratio, 1.015; 95% confidence interval, 1.002–1.028; *p* = 0.025), age (*p* = 0.010), and UPCR (*p* = 0.046) remained independently associated with HPAS. Log-transformed acrolein was positively correlated with bilateral CAVI (all *p* < 0.001) and log-transformed UPCR (*p* < 0.001) but negatively correlated with the estimated glomerular filtration rate (*p* = 0.001). Conclusions: Elevated serum acrolein is independently associated with HPAS in non-dialysis CKD stages 3–5.

## 1. Introduction

Cardiovascular disease is the leading cause of morbidity and mortality in patients with chronic kidney disease (CKD) [1,2]. Among the vascular abnormalities observed in CKD, arterial stiffness is a significant feature of accelerated vascular aging that has been associated with adverse cardiovascular outcomes [3,4]. The cardio-ankle vascular index (CAVI) is a noninvasive parameter used to assess arterial stiffness that is less influenced by blood pressure at the time of measurement than conventional pulse-wave velocity [5,6]. Elevated CAVI has been associated with cardiovascular risk in patients with CKD, supporting its potential value in pre-dialysis vascular assessment [4,7].

Acrolein, a reactive aldehyde produced during lipid peroxidation and polyamine metabolism, has been implicated in oxidative stress and endothelial injury [8,9,10,11]. Patients with impaired renal function tend to accumulate acrolein because of reduced clearance and increased oxidative burden [9,12]. Studies have found associations between acrolein exposure or its metabolites and cardiovascular risk or renal dysfunction, mainly in general or non-CKD populations [12,13]. However, clinical data on the relationship between circulating acrolein levels and arterial stiffness in patients with non-dialysis CKD are still limited.

Given the high cardiovascular burden in CKD, potentially clinically relevant biomarkers of vascular injury must be identified. In this cross-sectional study, we examined the association between serum acrolein levels and high peripheral arterial stiffness (HPAS) as assessed by CAVI in adults with stage 3–5 non-dialysis CKD. We also evaluated the association between serum acrolein and PAS after adjusting for conventional clinical factors.

## 2. Results

### 2.1. Baseline Characteristics

The study population consisted of 204 patients with stage 3–5 non-dialysis CKD, which was divided into a control group (*n* = 114) and an HPAS group (*n* = 90). The baseline biochemical and demographic characteristics of the patients are summarized in Table 1. The mean age of the participants was 68.55 ± 11.92 years, and 41.2% had diabetes mellitus (DM). Compared with the control group, the HPAS group was significantly older (*p* = 0.023) and had a significantly higher prevalence of DM (*p* = 0.047). Furthermore, the HPAS group had a significantly higher spot urine protein-to-creatinine ratio (UPCR, *p* = 0.046) and significantly higher levels of fasting glucose (*p* = 0.041), serum acrolein (*p* = 0.008), and interleukin-6 (IL-6, *p* = 0.025). The two groups did not differ significantly in sex, hypertension prevalence, or CKD stage distribution.

### 2.2. Independent Predictors of High Peripheral Arterial Stiffness

Multivariable logistic regression analysis, adjusted for significant factors with *p* < 0.05 (including age, DM, fasting glucose, UPCR, IL-6, and acrolein; Table 1), identified age (odds ratio [OR]: 1.039 per 1-year increase in age; 95% confidence interval [CI]: 1.011–1.068; *p* = 0.007), serum acrolein level (OR: 1.016 per 1-ng/mL increase; 95% CI: 1.003–1.028; *p* = 0.013), and spot UPCR (OR: 1.486 per 1-g increase; 95% CI: 1.035–2.133; *p* = 0.032) as independent predictors of HPAS in this cohort (Table 2). All variance inflation factors were <2.5, indicating no concern for multicollinearity.

### 2.3. Correlation Analysis and Multivariable Determinants of Left and Right CAVI by CKD Stage

In multivariable linear regression analysis, adjusted for significant factors with *p* < 0.05 in Table 1, stratified by CKD stage for left CAVI, older age (*p* = 0.046) and higher log acrolein (*p* = 0.007) were independently associated with higher left CAVI in stage 3 CKD, whereas age (*p* = 0.007) and log acrolein (*p* = 0.002) remained significant independent correlates in stage 4 CKD. In stage 5 CKD, only log acrolein (*p* = 0.040) remained significantly associated with left CAVI (Table 3).

In multivariable linear regression analysis, adjusted for significant factors with *p* < 0.05 in Table 1 and stratified by CKD stage, higher log acrolein (*p* = 0.015) was independently associated with higher right CAVI in stage 3 CKD. In stage 4 CKD, age (*p* = 0.001), log UPCR (*p* = 0.043), and log acrolein (*p* = 0.006) were all independently associated with higher right CAVI. In stage 5 CKD, log acrolein (*p* = 0.028) was the only significant independent correlate of right CAVI (Table 4). Overall, log acrolein was consistently positively associated with both left and right CAVI across CKD stages, supporting its potential link with HPAS in patients with stage 3–5 non-dialysis CKD.

### 2.4. Spearman’s Correlation Analysis

In Spearman’s correlation analysis (Table 5), log-acrolein levels were positively correlated with both left CAVI (*r* = 0.322, *p* < 0.001) and right CAVI (*r* = 0.289, *p* < 0.001). Furthermore, log-acrolein levels were significantly positively correlated with log-blood urea nitrogen (*r* = 0.148, *p* = 0.035), log-creatinine (*r* = 0.218, *p* = 0.002), log-UPCR (*r* = 0.246, *p* < 0.001), log-glucose (*r* = 0.186, *p* = 0.008), and log-IL-6 (*r* = 0.212, *p* = 0.002) levels. However, they were negatively correlated with the estimated glomerular filtration rate (eGFR, *r* = −0.224, *p* = 0.001) and hemoglobin (*r* = −0.165, *p* = 0.019) levels. Both the left and right CAVI were positively correlated with age (*r* = 0.163, *p* = 0.020 and *r* = 0.199, *p* = 0.004, respectively), log UPCR (*r* = 0.163, *p* = 0.020 and *r* = 0.198, *p* = 0.005), and log IL-6 (*r* = 0.219, *p* = 0.002 and *r* = 0.161, *p* = 0.021).

## 3. Discussion

This cross-sectional study of adults with stage 3–5 non-dialysis CKD confirmed an association between higher serum acrolein levels and higher CAVI values with the presence of HPAS. Older age and higher spot UPCR were independently associated with HPAS in multivariate analysis. These findings indicate an association between serum acrolein levels and increased arterial stiffness in patients with non-dialysis CKD. Beyond its well-recognized role as an environmental pollutant, acrolein is also an endogenous uremic toxin, a byproduct of oxidative stress and lipid peroxidation that accumulates with declining renal clearance [9,14]. Our findings revealed a significant negative correlation between acrolein and eGFR, suggesting that a uremic environment may impair aldehyde clearance while enhancing endogenous acrolein production.

Arterial stiffness is emerging as an important manifestation of vascular injury in CKD and is associated with adverse cardiovascular outcomes [3,4]. CAVI has been used as a pressure-independent marker of peripheral arterial stiffness (PAS) that may be useful for vascular risk assessment in the non-dialysis CKD population [5,6]. In the present study, patients with HPAS were older and had higher fasting glucose, IL-6, UPCR, and serum acrolein levels than those without PAS. These findings are generally consistent with the multifactorial development of arterial stiffness in CKD, which reflects the combined effects of aging, metabolic disturbances, inflammation, and renal injury.

Acrolein promotes protein carbonylation and suppresses endothelial nitric oxide synthase (eNOS) activity, thereby reducing nitric oxide bioavailability and impairing endothelium-dependent vasodilation [15,16]. Therefore, acrolein was associated with the development of permanent arterial structural stiffness and vascular calcification [10,17]. The independent predictive value of acrolein for HPAS, along with age and UPCR, persisted after adjustment for inflammatory markers, such as IL-6, underscoring a distinct inflammation-independent pathway of vascular injury. In our cohort, serum acrolein levels were negatively correlated with eGFR and positively correlated with UPCR, IL-6, and bilateral CAVI values. These observations substantiate the association between acrolein and both declining renal function and vascular injury in patients with CKD.

The association between systemic toxins and vascular health in renal impairment is increasingly recognized as a critical determinant of cardiovascular prognosis. Previous studies have established a correlation between elevated acrolein levels and an increased risk of cardiovascular disease [13]. However, there is a critical need to bridge the gap between aldehyde-induced molecular damage and the clinical manifestation of arterial stiffness in patients with non-dialysis CKD. Our study addresses this gap by demonstrating a direct and independent association between serum acrolein levels and CAVI-derived HPAS.

Experimental evidence suggests that acrolein may contribute to arterial stiffening through oxidative stress, protein carbonylation, and impaired endothelial nitric oxide signaling [11,18,19,20,21,22]. These processes may promote endothelial injury, reduce nitric oxide bioavailability, and contribute to vascular wall remodeling. In the uremic environment, where oxidative stress and antioxidant defense imbalance are common, acrolein accumulation may further amplify vascular injury and arterial stiffening. In addition, acrolein has been reported to stimulate inflammatory responses, including IL-6 production, in experimental models [23,24]. In our cohort, serum acrolein was positively correlated with IL-6, suggesting a possible link between acrolein accumulation and inflammation in patients with CKD.

Our findings expand on the existing literature in different domains. Regarding the methodology of acrolein measurement, the seminal work by DeJarnett et al. [13] in the Louisville Healthy Heart Study assessed acrolein exposure indirectly using urinary metabolite 3-hydroxypropylmercapturic acid (3-HPMA) in 211 subjects from the general population with moderate-to-high cardiovascular risk. The study found associations between urinary 3-HPMA levels and platelet–leukocyte aggregates, as well as with the Framingham Risk Score. However, that study did not evaluate arterial stiffness as an outcome and was conducted in a non-CKD population. Furthermore, it quantified urinary metabolites but did not measure serum aldehyde directly, a methodological distinction that limits its comparability to the uremic context. Similarly, a large-scale cross-sectional analysis of NHANES data (*n* = 4951) confirmed an inverse association between urinary acrolein metabolites and eGFR [12], providing epidemiological evidence for interactions between acrolein and kidney function. However, it also used indirect metabolite surrogates and did not specifically assess vascular outcomes in the pre-dialysis CKD population. Our study demonstrates serum acrolein quantification as an independent predictor of pressure-independent HPAS, as measured by CAVI in a non-dialysis CKD cohort. Notably, the independent predictive value of acrolein in multivariable logistic regression, after adjusting for IL-6 and other traditional risk factors, suggests that it may be linked to HPAS through mechanisms not fully captured by existing biomarkers, supporting its incremental clinical value within a multi-marker risk stratification framework rather than as a stand-alone diagnostic test.

The implications with respect to preventing major adverse cardiovascular events (MACEs) are considerable. Patients with CKD who progress to dialysis carry a cardiovascular mortality risk approximately 10–30 times higher than that of the general population [1,2]. A substantial proportion of this excess risk is attributable to arterial stiffness-driven left ventricular afterload, which is accelerated in a uremic oxidative environment [4]. Identifying patients with subclinical acrolein-mediated vascular injury at CKD stages 3–5—crucially. before the onset of symptomatic cardiovascular disease or dialysis dependence—is therefore critical. Targeted strategies ranging from thiol-based antioxidant therapy and dietary aldehyde restriction to optimized convective dialysis modalities in the transition to renal replacement therapy may attenuate the trajectory of acrolein-driven vascular injury and reduce the downstream MACE burden [24,25,26,27]. Beyond biomarker validation, the translational implications of our findings extend to therapeutic intervention. The references cited herein indicate that thiol-based antioxidants (e.g., N-acetylcysteine), dietary polyphenols with aldehyde-scavenging properties, and convective dialysis modalities may attenuate acrolein-mediated vascular toxicity. Whether these interventions can reduce CAVI and subsequently lower the risk of MACE in pre-dialysis CKD patients remains to be confirmed in future prospective studies. Future interventional studies using serial CAVI measurement as a pre-specified endpoint are needed. These studies would help determine whether targeting acrolein represents a viable strategy for reducing vascular risk in CKD.

Several limitations of this study warrant consideration in interpreting the findings. First, the cross-sectional design precluded the establishment of temporal causality between elevated serum acrolein levels and the development of HPAS. Longitudinal cohort studies with serial acrolein and CAVI measurements must be conducted to establish directionality and assess whether acrolein trajectories predict incident cardiovascular events or CKD progression. Second, this study was conducted at a single tertiary center, and the relatively modest sample size may limit the generalizability of our findings to CKD populations across different ethnic backgrounds, dietary habits, and healthcare systems. Third, potential confounders of acrolein levels, including smoking status [28], dietary acrolein intake [29] (e.g., from heated cooking oils), and occupational environmental exposures [29], were not evaluated, which may have introduced residual confounding into the association between serum acrolein and vascular stiffness. Fourth, although CAVI reflects pressure-independent intrinsic arterial wall stiffness [30], it has inherent technical limitations, including potential variability introduced by cardiac arrhythmias, severe aortic regurgitation, and significant peripheral arterial occlusive disease. These conditions were not systematically excluded from all participants, potentially introducing measurement heterogeneity. Fifth, data on several clinically important determinants of arterial stiffness were not systematically collected. These include smoking status, active pharmacological treatments known to influence arterial stiffness, such as renin–angiotensin–aldosterone system inhibitors, statins, and certain antidiabetic agents; duration of diabetes; and the use of calcium or vitamin D supplementation. Such factors are clinically relevant in CKD populations and may independently affect CAVI values. Their absence from the multivariable model may therefore have introduced residual confounding. Future prospective studies should incorporate comprehensive data on smoking exposure, medication use, diabetes duration, and mineral-bone metabolism-related treatments to address this limitation. Sixth, serum acrolein was measured using a commercial enzyme-linked immunosorbent assay that does not distinguish free acrolein from protein-bound acrolein or acrolein-derived adducts. Therefore, the measured values should be interpreted as serum immunoreactive acrolein rather than a specific molecular fraction of circulating acrolein. Further studies using mass spectrometry or assays specific for acrolein protein adducts are needed to clarify which molecular form is associated with arterial stiffness. In addition, evaluating acrolein simultaneously with established vascular biomarkers, such as fibroblast growth factor-23, symmetric dimethylarginine, or high-sensitivity C-reactive protein, may enhance predictive performance. Prospective studies with larger, more diverse CKD populations are warranted to confirm these findings and evaluate the association between serum acrolein and longitudinal vascular or cardiovascular outcomes.

## 4. Conclusions

In conclusion, higher serum acrolein levels were associated with HPAS and higher CAVI values in adults with stage 3–5 non-dialysis CKD. However, while these findings suggest a possible association between circulating acrolein and vascular stiffness in CKD, they should be interpreted cautiously, given the cross-sectional study design and potential residual confounding. Further prospective and mechanistic studies are required to clarify the clinical significance of this association.

## 5. Materials and Methods

### 5.1. Participants

Between January and June 2021, we screened and enrolled 204 adult patients with stage 3–5 non-dialysis CKD from the renal outpatient clinic of a medical center in Hualien, Taiwan. To ensure diagnostic accuracy, CKD was defined by two separate eGFR measurements taken at least 3 months apart and calculated using the Chronic Kidney Disease Epidemiology Collaboration equation. Participants were categorized according to the Kidney Disease Outcomes Quality Initiative criteria as follows: CKD stage 3 (eGFR 60–30 mL/min/1.73 m^2^), CKD stage 4 (eGFR 29–15 mL/min/1.73 m^2^), and CKD stage 5 (eGFR < 15 mL/min/1.73 m^2^). While all participants were supervised under a standard multidisciplinary care program emphasizing salt and protein restriction alongside the avoidance of nephrotoxins, we excluded individuals who presented with malignancies, chronic inflammatory conditions, chronic obstructive lung disease, heart failure, liver cirrhosis, stroke, limb amputation, and acute coronary syndrome. Patients already requiring dialysis and those scheduled for a kidney transplant within 6 months were also excluded. Regarding comorbidities, DM was defined as a fasting glucose level ≥126 mg/dL or the use of antidiabetic medication, while hypertension was defined as a blood pressure reading ≥140/90 mmHg or active use of antihypertensive agents. The Institutional Review Board of Hualien Tzu Chi Hospital approved the study protocol (IRB108-219-A), and every participant provided written informed consent prior to patient data collection.

### 5.2. Anthropometric Analysis

Anthropometric measurements were performed after an overnight fast. Body weight and height were recorded to the nearest 0.5 kg and 0.5 cm, respectively, and body mass index was calculated as weight (kg) divided by the square of height (m^2^).

### 5.3. Biochemical Investigations

For blood analysis, 5 mL fasting samples were collected. Hemoglobin was assessed using a Sysmex XS-1000i instrument (Sysmex America, Mundelein, IL, USA) and 0.5 mL of blood. The remaining blood was centrifuged at 3000× *g* to isolate serum for analysis using a Siemens Advia 1800 autoanalyzer (Siemens Healthcare GmbH, Erlangen, Germany). We evaluated a broad panel of serum biochemical parameters, including total cholesterol, triglycerides, low-density lipoprotein cholesterol (LDL-C), blood urea nitrogen, creatinine, albumin, fasting glucose, calcium, and phosphorus. Furthermore, a random spot urine sample was collected to measure the UPCR. Serum acrolein (MyBioSource, San Diego, CA, USA; Cat. No. MBS286800) and IL-6 (Abcam, Waltham, MA, USA; Cat. No. ab46042) were quantified using specialized enzyme-linked immunosorbent assay kits. The intra- and inter-assay coefficients of variation were 5.6% and 4.8% for acrolein and 3.8% and 5.0% for IL-6, respectively.

### 5.4. Blood Pressure and Carotid-Ankle Vascular Index Measurements

Blood pressure was measured after participants had been resting in the supine position for 10 min in a quiet, comfortable environment. Measurements were performed by trained staff using a calibrated mercury sphygmomanometer with an appropriately sized cuff. CAVI values were assessed using a waveform analyzer (VaSera VS-1000, Fukuda Denshi, Tokyo, Japan) [31]. After 10 min of rest in the supine position, the device automatically calculated CAVI based on electrocardiographic, phonocardiographic, and limb blood pressure recordings. High CAVI was defined as ≥9.0, in accordance with the expert consensus proposed by the Physiological Diagnosis Criteria for Vascular Failure Committee of the Japan Society for Vascular Failure [32]. The primary binary classification of HPAS was based on the higher CAVI value, defined as either right or left CAVI ≥ 9.0. Patients with bilateral CAVI < 9.0 were classified into the control group. Right- and left-sided CAVI values were also reported separately as continuous measures to assess internal consistency and to determine whether the association between serum acrolein and CAVI was consistent across laterality.

### 5.5. Statistical Analyses

To ensure the robustness of our findings, we first evaluated the normality of all continuous data using the Kolmogorov–Smirnov test. Variables with a normal distribution are expressed as mean ± standard deviation, whereas those with skewed distributions are expressed as medians and interquartile ranges. For group comparisons, we used Student’s two-tailed independent *t*-test for parametric data and the Mann–Whitney U test for non-parametric data. Categorical data are expressed as frequencies and percentages and were analyzed using the chi-square test. To identify potential predictors of high CAVI (HPAS group), any significant variable in univariable analysis was subsequently included in the multivariable logistic regression model. Several clinical markers—specifically, total cholesterol, triglycerides, LDL-C, eGFR, UPCR, albumin, fasting glucose, and serum acrolein—exhibited significant skewness and were log-transformed before further analysis. Multivariable linear regression analyses were performed using the forced-entry method. Within each CKD stage, all prespecified covariates that were significant in univariable analysis between the HPAS and control groups were entered simultaneously into the model. We utilized Spearman’s rank correlation coefficients to explore the relationships between these clinical parameters and vascular markers (left/right CAVI and log acrolein). All statistical analyses were performed using SPSS for Windows (version 25.0; IBM Corp., Armonk, NY, USA) and R statistical software (version 4.2.2; R Foundation for Statistical Computing, Vienna, Austria), with a significance level of *p* < 0.05.

## Figures and Tables

**Table 1:** Demographic, clinical, and biochemical characteristics of the study population according to high peripheral arterial stiffness status.

Parameters	All Patients (*n* = 204)	Control Group (*n* = 114)	HPAS Group (*n* = 90)	*p* Value
Age (years)	68.55 ± 11.92	66.88 ± 12.60	70.68 ± 10.68	0.023 *
Height (cm)	160.22 ± 8.33	160.36 ± 8.66	160.04 ± 7.94	0.789
Body weight (kg)	67.60 ± 12.82	68.47 ± 14.01	66.50 ± 11.11	0.277
Body mass index (kg/m^2^)	26.28 ± 4.26	26.54 ± 4.55	25.94 ± 3.85	0.320
Left CAVI	8.16 ± 2.91	6.32 ± 1.82	10.50 ± 2.27	<0.001 *
Right CAVI	8.39 ± 2.90	6.39 ± 1.80	10.92 ± 1.89	<0.001 *
SBP (mmHg)	137.38 ± 22.53	137.68 ± 22.98	136.99 ± 22.07	0.827
DBP (mmHg)	65.00 (58.25–74.00)	65.00 (58.00–76.00)	64.00 (59.00–71.00)	0.545
Total cholesterol (mg/dL)	151.56 ± 43.85	152.08 ± 43.75	150.91 ± 44.21	0.830
Triglyceride (mg/dL)	131.50 (98.50–188.00)	130.00 (97.75–185.00)	140.00 (103.00–195.25)	0.463
LDL-C (mg/dL)	78.46 ± 32.04	80.15 ± 32.51	76.31 ± 31.49	0.397
Fasting glucose (mg/dL)	111.50 (97.25–134.00)	109.00 (98.75–127.25)	122.00 (96.00–143.50)	0.041 *
Blood urea nitrogen (mg/dL)	38.00 (27.00–52.00)	38.00 (29.00–51.25)	36.50 (25.00–52.25)	0.632
Creatinine (mg/dL)	2.42 (1.90–3.25)	2.57 (1.89–3.39)	2.36 (1.91–3.11)	0.428
eGFR (mL/min)	26.88 ± 12.10	26.57 ± 12.37	27.27 ± 11.80	0.680
Spot UPCR (g/g)	1.12 (0.50–1.64)	1.07 (0.41–1.52)	1.19 (0.59–2.03)	0.046 *
Albumin (g/dL)	4.00 (3.80–4.20)	4.00 (3.68–4.20)	4.10 (3.90–4.20)	0.205
Total calcium (mg/dL)	9.29 ± 0.44	9.28 ± 0.41	9.30 ± 0.47	0.721
Phosphorus (mg/dL)	3.75 ± 0.72	3.77 ± 0.72	3.72 ± 0.73	0.606
Hemoglobin (g/dL)	11.44 ± 2.11	11.35 ± 2.09	11.55 ± 2.14	0.500
Acrolein (ng/mL)	38.22 (27.43–58.68)	36.12 (24.81–52.53)	41.62 (28.89–70.61)	0.008 *
IL-6 (pg/mL)	5.63 (3.72–8.98)	5.27 (3.35–8.40)	6.18 (4.24–9.69)	0.025 *
Female, *n* (%)	86 (42.2)	52 (45.6)	34 (37.8)	0.260
Diabetes mellitus, *n* (%)	84 (41.2)	40 (35.1)	44 (48.9)	0.047 *
Hypertension, *n* (%)	114 (55.9)	62 (54.4)	52 (57.8)	0.628
Glomerulonephritis, *n* (%)	59 (28.9)	37 (32.5)	22 (24.4)	0.210
CKD stage 3, *n* (%)	68 (33.3)	34 (29.8)	34 (37.8)	0.310
CKD stage 4, *n* (%)	101 (49.5)	57 (50.0)	44 (48.9)	
CKD stage 5, *n* (%)	35 (17.2)	23 (20.2)	12 (13.3)	

Continuous variables are presented as the mean ± standard deviation and were compared using Student’s *t*-test. Variables with a non-normal distribution are presented as the median (interquartile range) and were compared using the Mann–Whitney *U* test. Categorical variables are presented as numbers (%) and were analyzed using the chi-square test. HPAS, high peripheral arterial stiffness; CAVI, cardio-ankle vascular index; SBP, systolic blood pressure; DBP, diastolic blood pressure; LDL-C, low-density lipoprotein cholesterol; eGFR, estimated glomerular filtration rate; UPCR, urine protein-to-creatinine ratio; IL-6, interleukin-6; CKD, chronic kidney disease. * *p* < 0.05 was considered statistically significant.

**Table 2:** Multivariable logistic regression analysis of factors associated with high peripheral arterial stiffness.

Determinant	Odds Ratio	95% Confidence Interval	*p* Value
Acrolein, 1 ng/mL	1.016	1.003—1.028	0.013 *
Age, 1 year	1.039	1.011—1.068	0.007 *
Spot UPCR, 1 g/g	1.486	1.035—2.133	0.032 *
Diabetes mellitus, present	1.023	0.386—2.711	0.964
Fasting glucose, 1 mg/dL	1.011	0.992—1.030	0.256
IL-6, 1 pg/mL	1.024	0.973—1.077	0.368

Variables with *p* < 0.05 in Table 1, including diabetes mellitus, age, fasting glucose, spot UPCR, IL-6, and acrolein, were entered into the multivariable logistic regression analysis. OR, odds ratio; CI, confidence interval; UPCR, urine protein-to-creatinine ratio; IL-6, interleukin-6. * *p* < 0.05 was considered statistically significant.

**Table 3:** Multivariable linear regression for the left carotid-ankle vascular index by CKD stage.

CKD Stage	Predictor	B	SE	β	t	95% CI	*p* Value
Stage 3	Age	0.080	0.039	0.240	2.037	0.001 to 0.158	0.046 *
	Log UPCR	0.529	0.935	0.071	0.565	−1.341 to 2.398	0.574
	Log glucose	3.138	4.279	0.089	0.733	−5.415 to 11.692	0.466
	Log IL-6	1.868	1.275	0.188	1.465	−0.681 to 4.417	0.148
	Log acrolein	4.309	1.553	0.352	2.774	1.204 to 7.413	0.007 *
Stage 4	Age	0.054	0.019	0.275	2.771	0.015 to 0.092	0.007 *
	Log UPCR	0.948	0.740	0.131	1.282	−0.521 to 2.417	0.203
	Log glucose	−0.112	2.976	−0.004	−0.038	−6.021 to 5.796	0.970
	Log IL-6	0.792	0.715	0.116	1.109	−0.626 to 2.211	0.270
	Log acrolein	3.483	1.113	0.315	3.129	1.274 to 5.693	0.002 *
Stage 5	Age	0.010	0.043	0.038	0.225	−0.079 to 0.099	0.824
	Log UPCR	1.022	1.877	0.099	0.544	−2.818 to 4.861	0.590
	Log glucose	−1.833	5.330	−0.072	−0.344	−12.735 to 9.068	0.733
	Log IL-6	2.393	1.493	0.271	1.603	−0.661 to 5.446	0.120
	Log acrolein	4.081	1.896	0.420	2.153	0.204 to 7.958	0.040 *

Data for glucose, UPCR, IL-6, and acrolein showed skewed distributions and were therefore log-transformed before analysis. We stratified analyses by CKD stage and treated the left carotid-ankle vascular index (left CAVI) as a continuous dependent variable. Covariates were prespecified based on clinical relevance and included age, log UPCR, log glucose, log IL-6, and log acrolein. Within each CKD stage, predictors were entered into a forced-entry multiple linear regression. We report unstandardized coefficients (B) with standard errors (SE), standardized coefficients (β), t-statistics, two-sided *p*-values, and 95% confidence intervals (CI). UPCR, urine protein-to-creatinine ratio; IL-6, interleukin-6. * *p* < 0.05 was considered statistically significant. Coefficient of determination (R^2^) values were 0.227, 0.191, and 0.231 for CKD stages 3, 4, and 5, respectively; the corresponding adjusted R^2^ values were 0.165, 0.148, and 0.098, and the overall model *p*-values were 0.006, 0.001, and 0.157.

**Table 4:** Multivariable linear regression for the right carotid-ankle vascular index by CKD stage.

CKD Stage	Predictor	B	SE	β	t	95% CI	*p* Value
Stage 3	Age	0.054	0.039	0.170	1.391	−0.023 to 0.131	0.169
	Log UPCR	1.719	0.921	0.238	1.866	−0.122 to 3.560	0.067
	Log glucose	−1.665	4.214	−0.050	−0.395	−10.088 to 6.758	0.694
	Log IL-6	0.541	1.256	0.052	0.430	−1.970 to 3.051	0.668
	Log acrolein	3.821	1.529	0.293	2.498	0.763 to 6.878	0.015 *
Stage 4	Age	0.067	0.020	0.319	3.382	0.028 to 0.106	0.001 *
	Log UPCR	1.541	0.753	0.196	2.048	0.047 to 3.035	0.043 *
	Log glucose	1.555	3.026	0.048	0.514	−4.453 to 7.563	0.609
	Log IL-6	0.428	0.727	0.056	0.589	−1.015 to 1.871	0.557
	Log acrolein	3.200	1.132	0.272	2.827	0.953 to 5.448	0.006 *
Stage 5	Age	0.038	0.040	0.154	0.960	−0.043 to 0.120	0.345
	Log UPCR	2.592	1.727	0.258	1.501	−0.939 to 6.123	0.144
	Log glucose	−1.913	4.902	−0.077	−0.390	−11.939 to 8.113	0.699
	Log IL-6	2.300	1.373	0.267	1.675	−0.508 to 5.109	0.105
	Log acrolein	4.034	1.743	0.426	2.314	0.469 to 7.600	0.028 *

Data for glucose, UPCR, IL-6, and acrolein showed skewed distributions and were therefore log-transformed before analysis. We stratified analyses by CKD stage and treated the right carotid-ankle vascular index (right CAVI) as a continuous dependent variable. Covariates were prespecified based on clinical relevance and included age, log UPCR, log glucose, log IL-6, and log acrolein. Within each CKD stage, predictors were entered into a forced-entry multiple linear regression. We report unstandardized coefficients (B) with standard errors (SE), standardized coefficients (β), t-statistics, two-sided *p*-values, and 95% confidence intervals (CI). UPCR, urine protein-to-creatinine ratio; IL-6, interleukin-6. * *p* < 0.05 was considered statistically significant. Coefficient of determination (R^2^) values were 0.212, 0.210, and 0.316 for CKD stages 3, 4, and 5, respectively; the corresponding adjusted R^2^ values were 0.148, 0.169, and 0.198, and the overall model *p*-values were 0.010, <0.001, and 0.041.

**Table 5:** Spearman’s correlation analysis of left CAVI, right CAVI, and acrolein with clinical variables.

Variables	Left CAVI		Right CAVI		Acrolein (ng/mL)	
	Spearman’s Coefficient of Correlation	*p* Value	Spearman’s Coefficient of Correlation	*p* Value	Spearman’s Coefficient of Correlation	*p* Value
Age (years)	0.163	0.020 *	0.199	0.004 *	–0.122	0.081
Body mass index (kg/m^2^)	–0.062	0.380	0.114	0.103	–0.004	0.950
Left CAVI	—	—	0.882	<0.001 *	0.322	<0.001 *
Right CAVI	0.882	<0.001 *	—	—	0.289	<0.001 *
Acrolein (ng/mL)	0.322	<0.001 *	0.289	<0.001 *	—	—
SBP (mmHg)	–0.100	0.154	–0.067	0.341	–0.089	0.207
DBP (mmHg)	–0.088	0.209	–0.098	0.161	0.012	0.870
TCH (mg/dL)	–0.007	0.922	0.002	0.982	0.020	0.775
Triglyceride (mg/dL)	0.009	0.894	0.003	0.963	0.009	0.895
LDL-C (mg/dL)	–0.070	0.321	–0.075	0.284	0.027	0.700
BUN (mg/dL)	0.017	0.810	–0.028	0.694	0.148	0.035 *
Creatinine (mg/dL)	0.005	0.942	–0.051	0.466	0.218	0.002 *
eGFR (mL/min)	–0.023	0.747	0.063	0.368	–0.224	0.001 *
UPCR (mg/g)	0.163	0.020 *	0.198	0.005 *	0.246	<0.001 *
Glucose (mg/dL)	0.072	0.303	0.085	0.225	0.186	0.008 *
Albumin (g/dL)	0.106	0.132	0.128	0.067	–0.034	0.631
Total calcium (mg/dL)	–0.054	0.445	0.047	0.505	–0.045	0.520
Phosphorus (mg/dL)	0.020	0.774	–0.032	0.655	0.120	0.087
Hemoglobin (g/dL)	0.004	0.952	0.045	0.518	–0.165	0.019 *
IL-6 (pg/mL)	0.219	0.002 *	0.161	0.021 *	0.212	0.002 *

Data analysis was performed using Spearman’s correlation. CAVI, cardio-ankle vascular index; SBP, systolic blood pressure; DBP, diastolic blood pressure; TCH, total cholesterol; LDL-C, low-density lipoprotein cholesterol; BUN, blood urea nitrogen; eGFR, estimated glomerular filtration rate; UPCR, urine protein-to-creatinine ratio; IL-6, interleukin 6. * *p* < 0.05 was considered statistically significant (two-tailed).

## Data Availability

The original contributions presented in this study are included in this article. Further inquiries can be directed to the corresponding author.

## Abbreviations

3-HPMA	3-hydroxypropylmercapturic acid
CAVI	Cardio-ankle vascular index
CKD	Chronic kidney disease
CKD-EPI	Chronic Kidney Disease Epidemiology Collaboration
DM	Diabetes mellitus
eNOS	Endothelial nitric oxide synthase
eGFR	Estimated glomerular filtration rate
HPAS	High peripheral arterial stiffness
IL-6	Interleukin-6
MACE	Major adverse cardiovascular event
PAS	Peripheral arterial stiffness
UPCR	Urine protein-to-creatinine ratio
